# Impact of Antigen Density on the Binding Mechanism of IgG Antibodies

**DOI:** 10.1038/s41598-017-03942-z

**Published:** 2017-06-19

**Authors:** Maya Hadzhieva, Anastas D. Pashov, Srinivas Kaveri, Sébastien Lacroix-Desmazes, Hugo Mouquet, Jordan D. Dimitrov

**Affiliations:** 1 Institute of Microbiology, Bulgarian Academy of Sciences, 1113 Sofia, Bulgaria; 2 Sorbonne Universités, UPMC Univ Paris 06, UMR_S 1138, Centre de Recherche des Cordeliers, 75006 Paris, France; 3 INSERM, UMR_S 1138, Centre de Recherche des Cordeliers, 75006 Paris, France; 4 Université Paris Descartes, Sorbonne Paris Cité, UMR_S 1138, Centre de Recherche des Cordeliers, 75006 Paris, France; 5 Laboratory of Humoral Response to Pathogens, Department of Immunology, Institut Pasteur, and INSERM U1222, 75015 Paris, France

## Abstract

The density and distribution pattern of epitopes at the surface of pathogens have a profound impact on immune responses. Although multiple lines of evidence highlight the significance of antigen surface density for antibody binding, a quantitative description of its effect on recognition mechanisms is missing. Here, we analyzed binding kinetics and thermodynamics of six HIV-1 neutralizing antibodies as a function of the surface density of envelope glycoprotein gp120. Antibodies that recognize gp120 with low to moderate binding affinity displayed the most pronounced sensitivity to variation in antigen density, with qualitative and substantial quantitative changes in the energetics of the binding process as revealed by non-equilibrium and equilibrium thermodynamic analyses. In contrast, the recognition of gp120 by the antibodies with the highest affinity was considerably less influenced by variations in antigen density. These data suggest that a lower affinity of antibodies permits higher dynamics during the antigen recognition process, which may have considerable functional repercussions. These findings contribute to a better understanding of the mechanisms of antigen recognition by antibodies. They are also of importance for apprehending the impact of antigen topology on immune-defense functions of antibodies.

## Introduction

Antibodies (Abs) are recognition molecules of the humoral adaptive immune response. Depending on the immunoglobulin class, Ab molecules possess between 2 and 10 identical antigen-binding sites. The binding valency of Abs has important functional implications during recognition of antigens. For example, IgM are a product of primary immune responses and usually have low affinity antigen binding sites^1^. However, the simultaneous engagement of their 10 antigen binding sites results in additive effect with a considerable increase in the overall functional affinity (avidity) of the molecule and elicitation of effector functions such as activation of the complement system^1^. Abs of the IgG class have two identical antigen-binding sites^2^. Bivalency of IgG contributes to crosslinking soluble antigens and forming high-molecular weight immune complexes – a prerequisite for activation of the complement system or crosslinking of Fc-gamma receptors^3–5^. In case of recognition of repetitive epitopes, as those found at the surfaces of pathogens, the bivalency of IgG contributes to enhancing the binding energy (affinity)^6^. Recognition of antigens by two Fab arms strongly depends on the intrinsic flexibility between the Fab and Fc portions of IgG molecule^7^. It has been proposed that some pathogens exploit the inability of IgG to form bivalent interactions as a selective advantage to evade Ab-mediated neutralization^8, 9^. Since the distance between the antigen binding sites of immunoglobulins is limited (150 Å in the case of IgG), the possibility for Abs to established bivalent interaction merely depends on the distribution density of epitopes at the pathogen surface^9^. Recent studies on human Abs have revealed two additional phenomena that strongly depend on the bivalency of IgGs, and on the special density of epitopes on pathogens surfaces. High-speed atomic force microscopy has demonstrated that IgG do not bind statically to antigenic surfaces but present with a dynamic stochastic behavior referred to as “IgG walking”^10^. Other studies have provided structural evidence about the formation of supramolecular complexes of IgG – called IgG hexamers – a process that depends on antigen recognition on surfaces^11, 12^. Importantly, non-covalently bound hexamers are the form of IgG functionally apt to direct activation of the complement system on surfaces^11, 13^.

Surface epitope density appears as a driving factor for all above-described observations; it controls Ab functions and thus modulates antibody-mediated immune responses. This is particularly true of humoral defense against viral infections as shown in the case of HIV where distance between gp120 spikes on viral envelope exceeds distance between the two “arms” of an IgG, and hence prevents efficient neutralization. Therefore, studies of the impact of density distribution of epitopes on the binding behavior of Abs are of uttermost relevance. In the present work, taking advantage of the availability of fully characterized human neutralizing monoclonal anti-gp120 IgG we investigated binding energetics of low and high affinity IgG1 Abs as a function of surface density for their target antigen - the gp120 glycoprotein of HIV-1.

## Results

### Binding kinetics of human Abs as a function of immobilization density of gp120 IIIB

**Figure 1:**
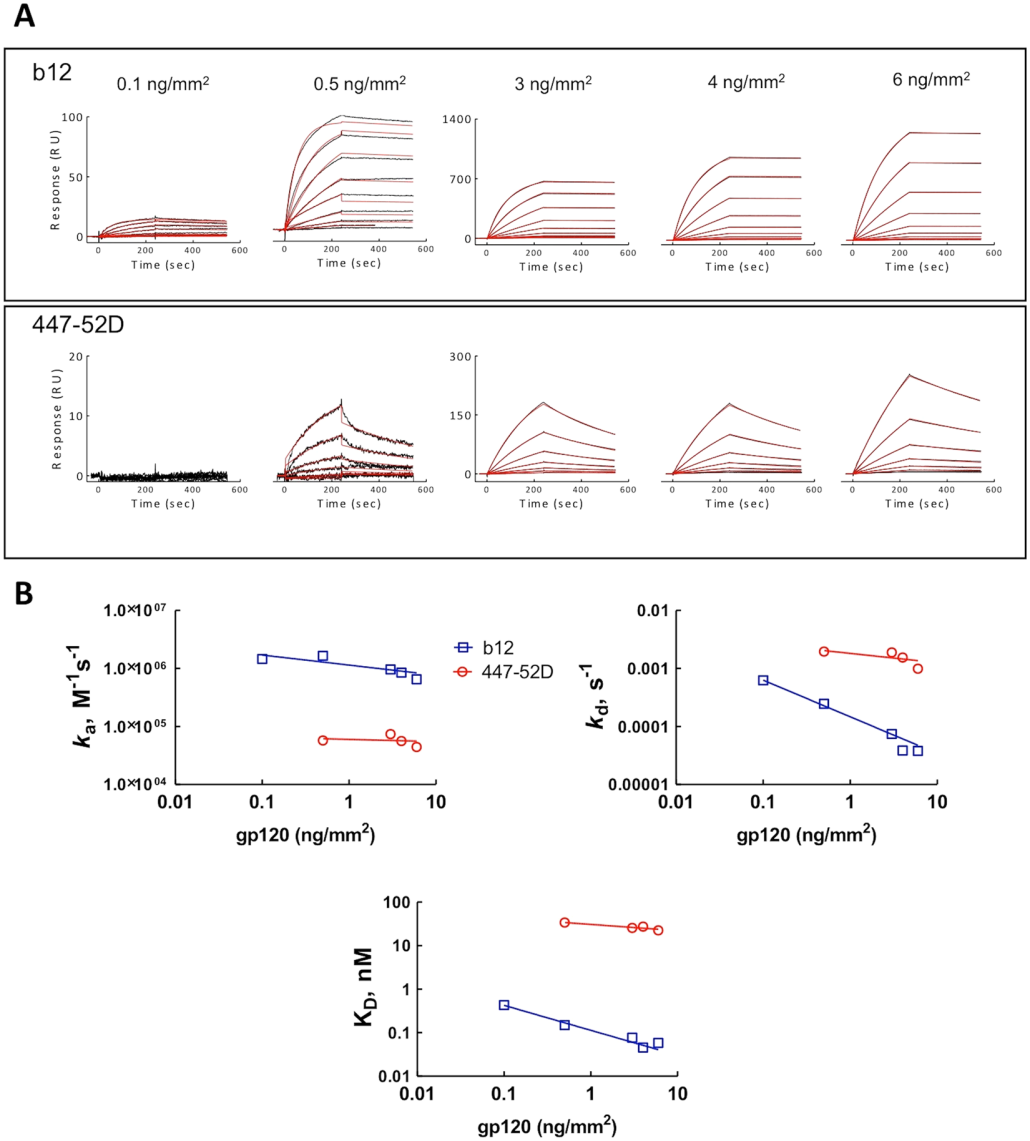
(**A**) Real time interaction profiles of Abs b12 and 447-52D as a function of immobilized density of gp120 (HIV-1 IIIB). The black line depicts the binding profiles obtained after injection of serial dilutions of b12 (25–0.048 nM) and 447-52D (50–0.097 nM). The red lines depict the fits of data obtained by global analyses using Biaevaluation software. The measurements were performed at 25 °C. (**B**) Effect of density of surface immobilized gp120 IIIB on the association rate constant; dissociation rate constant, and equilibrium dissociation constant of b12 (blue lines and symbols) and 447-52D (red line and symbols). The binding kinetics was assessed at 25 °C.

To provide insights on the effect of the density of surface immobilized antigen on the binding kinetics of Abs, we performed surface plasmon resonance (SPR)-based measurements (Fig. 1A). The immobilization of protein ligand to sensor chips followed an almost straightforward correlation of the resulting resonance response units (RU) with the amount of protein immobilized. Thus, response of 1000 RU was established to correspond to 1 ng/mm^2^ of surface-immobilized protein. We first covalently immobilized gp120 from the HIV-1 strain IIIB (clade B) to SPR-sensor chips with dextran surface with resulting protein densities of 0.1, 0.5, 3, 4 and 6 ng/mm^2^. Interactions of immobilized gp120 molecules with two human HIV-1-neutralizing Abs targeting two different epitopes, b12 and 447-52D, were first evaluated.

Real-time interaction profiles performed at 25 °C revealed that an increase of the immobilized density of gp120 correlated with an increase of the binding response to the sensor chip (Fig. 1A). For 447-52D, no binding was detected at a gp120 density of 0.1 ng/mm^2^. Evaluation of the binding kinetics of the Abs revealed that association rate constant at 25 °C of Ab 447-52D was not considerably influenced by variation of antigen density (Fig. 1B). Thus, the obtained *k*_a_ values were −5.7 × 10^4^ (±0.09) M^−1^ s^−1^ at gp120 density of 0.5 ng/mm^2^ and 4.4 × 10^4^ (±0.16) M^−1^ s^−1^ at gp120 density of 6 ng/mm^2^. Similarly, the values of dissociation constant *k*_d_ were only marginally influenced by the variation of density of gp120 (Fig. 1B). These results explain the small difference in the binding affinity of 447-52D at antigen density of 0.5 and 6 ng/mm^2^: K_D_ values of 34 and 22.5 nM, respectively (Fig. 1B). Interestingly, the effect of surface density of immobilized gp120 on kinetics of b12 was more pronounced (Fig. 1B). Indeed, *k*_a_ value of b12 decreased from 1.45 × 10^6^ (±0.02) M^−1^ s^−1^ to 0.65 × 10^6^ M^−1^ s^−1^, when estimated at a density of 0.1 and 6 ng/mm^2^, respectively. The values of *k*_d_ were considerably more affected, i.e., 6.26 × 10^−4^ (±0.05) s^−1^ and 3.8 × 10^−5^ (±0.01) s^−1^ at 0.1 and 6 ng/mm^2^, respectively (Fig. 1B). The differences in the binding kinetics account for the measured seven-fold increase in the binding affinity as the density of gp120 was elevated by sixty-fold (K_D_ values of 0.43 and 0.06 nM at 0.1 and 6 ng/mm^2^ density, respectively) (Fig. 1B). These data also confirmed that the affinity of b12 for gp120 IIIB is considerably higher (375-fold higher at density of 6 ng/mm^2^) than the affinity of 447-52D for the same antigen.

The observed reduction in the values of the association and dissociation rates of b12 as the density of antigen increase by sixty-fold may be explained by a contribution of mass transfer effects on the binding kinetics. Indeed, this phase effect is accompanied by a decrease in both kinetic rates constants and strongly depends on the density of the immobilized ligands^14–16^. Mass transfer influences considerably the interactions characterized by high association rates (as is the case of b12). In contrast, mass transfer negligibly influenced the binding kinetics of 447-52D.

### Binding thermodynamics of human Abs as a function of immobilization density of gp120 IIIB

**Figure 2:**
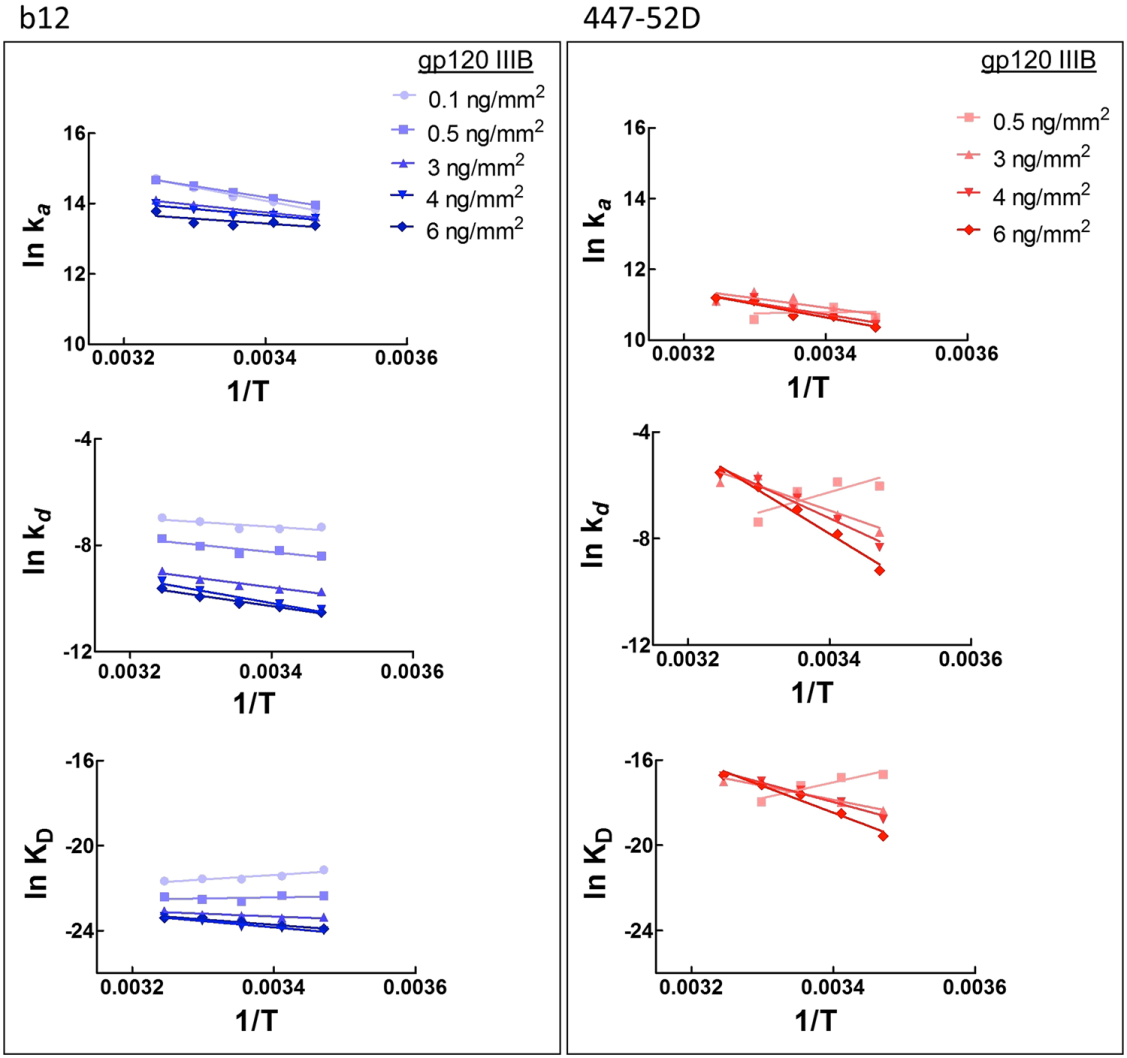
Temperature dependences of binding kinetics and affinity of Abs - b12 (different intensities of blue) and 447-52D (different intensities of red) for binding to various densities of immobilized gp120. The plots were generated by linear regression analyses. The Arrhenius plots were generated by linear regression analyses. Values of R^2^ of the presented fits are as follows: (1) b12, *k*_a_, 0.1 ng/mm^2^: 0.9857, 0.5 ng/mm^2^: 0.9997, 3 ng/mm^2^: 0.9596, 4 ng/mm^2^: 0.8657, 6 ng/mm^2^: 0.5564; *k*_d_, 0.1 ng/mm^2^: 0.6644, 0.5 ng/mm^2^: 0.8049, 3 ng/mm^2^: 0.9374, 4 ng/mm^2^: 0.9175, 6 ng/mm^2^: 0.9721; K_D_, 0.1 ng/mm^2^: 0.8475, 0.5 ng/mm^2^: 0.1463, 3 ng/mm^2^: 0.7897, 4 ng/mm^2^: 0.9423, 6 ng/mm^2^: 0.9417. (2) 447-52D, *k*_a_, 0.5 ng/mm^2^: 0.01112, 3 ng/mm^2^: 0.6398, 4 ng/mm^2^: 0.8680, 6 ng/mm^2^: 0.9407; *k*_d_, 0.5 ng/mm^2^: 0.6886, 3 ng/mm^2^: 0.8773, 4 ng/mm^2^: 0.9506, 6 ng/mm^2^: 0.9801; K_D_, 0.5 ng/mm^2^: 0.8916, 3 ng/mm^2^: 0.9502, 4 ng/mm^2^: 0.9644, 6 ng/mm^2^: 0.9666.

To gain mechanistic insight in the effect of variation of the antigen density on antigen recognition by monoclonal Abs, we investigated the activation and equilibrium thermodynamics of b12 and 447-52D. To this end, we measured the binding kinetics of Abs as a function of temperature. Arrhenius plots (natural logarithm of kinetic rate constants versus reciprocal temperature in Kelvin) showed that differences in the immobilization density of gp120 have only little impact on the temperature sensitivity of association and dissociation rate constants of the high affinity antibody b12 (deduced from the slopes of the plots) (Fig. 2). At all densities, the rate of association and dissociation weakly increased with augmentation of the temperature (Fig. 2). In contrast, a pronounced effect of antigen density on temperature sensitivity of rate constants was evidenced in the case of the low affinity Ab 447-52D. Thus, at an immobilization density of 0.5 ng/mm^2^, 447-52D had qualitatively different temperature dependence of *k*_a_ and *k*_d_ as compared to gp120 densities of 3, 4 and 6 ng/mm^2^. An increase of temperature resulted in a concomitant decrease in the rate constants of the Ab upon binding to low density of gp120 (Fig. 2). In addition, the steep slope of Arrhenius plots revealed that the temperature sensitivity of the dissociation rate at all gp120 densities was considerably higher for 447-52D as compared to the sensitivity of *k*_d_ of b12 (Fig. 2). Van’t Hoff plots depicted on Fig. 2 show that variation in reaction temperature slightly affected affinity of b12 at all antigen densities of gp120. In contrast, binding affinity was considerably more sensitive to changes in temperature for the interaction of 447-52D with its ligand (Fig. 2).

**Figure 3:**
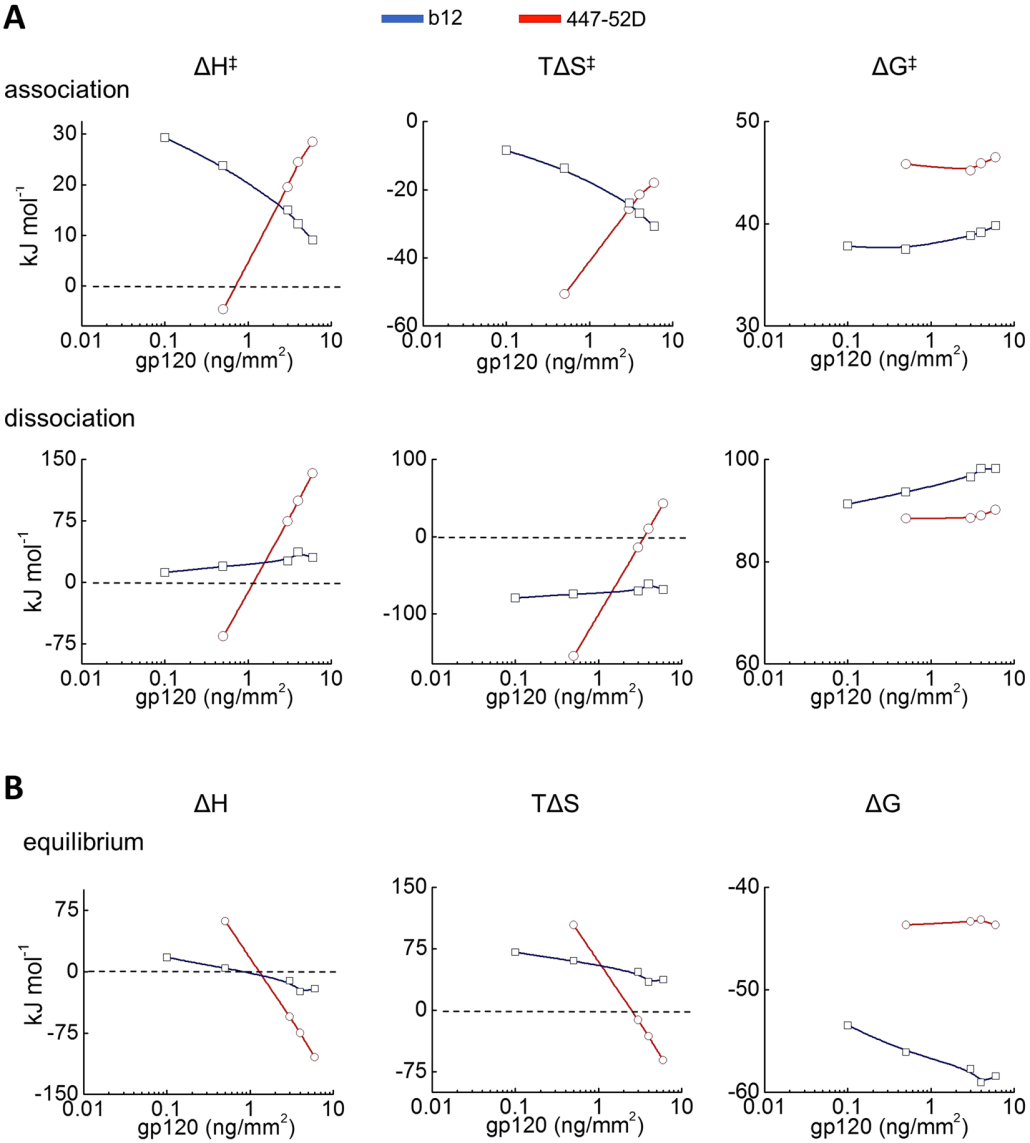
(**A**) Effect of density of surface-bound antigen on the activation thermodynamics of b12 (blue lines and symbols) and of 447-52D (red line and symbols). The values of the thermodynamic parameters were calculated by Eyring’s analyses using slopes of Arrhenius plots depicted on Fig. 2. All thermodynamic parameters were calculated at a reference temperature of 25 °C (293.7 K). (**B**) Effect of density of surface-bound antigen on the equilibrium thermodynamics of b12 (blue lines and symbols) and 447-52D (red line and symbols). The values of the thermodynamic parameters were evaluated as described in the experimental procedures. All equilibrium thermodynamic parameters were calculated at a reference temperature of 25 °C (293.7 K).

Furthermore, we evaluated the activation thermodynamics for the recognition of various gp120 densities by the selected Abs. An increase in the antigen density from 0.1 to 6 ng/mm^2^ had opposite effect on changes in enthalpy (ΔH) and entropy (TΔS) during associations of b12 and 447-52D (Fig. 3A). The changes in activation thermodynamics were more pronounced for 447-52D. In contrast to the relatively modest effect of gp120 density on the association thermodynamics for both Abs, antigen density had a profound effect on the changes in the thermodynamics of 447-52D during dissociation (Fig. 3A). Thus, an increase of gp120 surface density from 0.5 to 6 ng/mm^2^ resulted in qualitative and quantitative differences in changes in enthalpy and entropy. The resulting differences in the energy of more than 190 kJ mol^−1^ for both parameters were registered in the case of the low affinity Ab 447-52D. However, variations in antigen density had minimal effect (without qualitative changes) in the thermodynamic parameters of b12 (Fig. 3A). In contrast to the considerable changes detected in enthalpy and entropy, the changes in Gibbs free energy during association and dissociation were negligible for both Abs.

In addition, we evaluated the equilibrium thermodynamics of binding of Abs b12 and 447-52D (Fig. 3B). An increase in the surface density of gp120 resulted in qualitative changes in enthalpy for both b12 and 447-52D. Indeed, at low density, the binding process was characterized by favorable (for the overall binding affinity) changes in enthalpy (negative sign), whereas an increase in antigen density resulted in transition to unfavorable contribution of enthalpy (positive sign). It is noteworthy that quantitative extend of these alterations differed markedly for both Abs. Whereas an increase in the density of immobilized gp120 resulted in only 37 kJ mol^−1^ change in the equilibrium enthalpy for b12, it resulted in >160 kJ mol^−1^ difference in the case of 447-52D (Fig. 3B). Changes in equilibrium entropy as a function of antigen density were only minimal for b12. In contrast, considerable qualitative and quantitative effects were observed in the case of 447-52D. The entropy changes were favorable (positive sign) at low antigen densities and became highly unfavorable at densities ≥3 ng/mm^2^ (Fig. 3B). Taken together, the data from non-equilibrium and equilibrium thermodynamic analyses indicate that variations in antigen density have strong impact on the mechanism of antigen recognition by low affinity Abs. In contrast, high affinity Abs experience negligible effects that may well result from surface artifacts inherent to real time kinetics measurements (mass transport).

### Binding kinetics and thermodynamics of HIV-1 Abs specific for CD4-binding site as a function of immobilization density of gp120 CN54

Abs b12 and 447-52D recognize distinct regions of gp120 molecule. An epitope of b12 is localized in CD4-binding site on the gp120, whereas an epitope of 447-52D is localized in the V3 loop. To exclude the possibility that the observed differences in the recognition mechanism of low and high affinity Abs result from particular features of the binding regions, we extended our study by analyzing binding kinetics and thermodynamics of five HIV-1 neutralizing antibodies: HJ16, NIH45-46 G54W, b12, 2-1262 and CH103, that are specific for a common region on gp120 molecule – the CD4 binding site (CD4bs). Moreover, to control for strain specific effects of gp120, we used a variant of gp120 (CN54), from a different clade of HIV-1 (clade C). Kinetic analyses performed at 25 °C indicated that the selected Abs interact with gp120 with a large range of binding affinities (Table 1). Thus, at a moderate surface density of gp120 (1 ng/mm^2^), the difference in K_D_ values of the Ab with the lowest affinity (CH103) and of the Ab with the highest affinity (NIH45-46 G54W) was approximately 2800 folds (Table 1). The data also indicated that an increase of density of immobilized gp120 by *ca*. 24 folds results in only negligible changes in *k*_a_ constant of all Abs. However, *k*_d_ constant of all Abs was substantially affected (Table 1 and Supplemental Fig. 1), with a systematic decrease in the *k*_d_ constants with increases in immobilization density. The most affected was the dissociation of HJ16 for which the increase of gp120 density by 25 fold resulted in a reduction of *k*_d_ of approximately 150-fold.

**Table 1:** Kinetics of interactions of human monoclonal Abs with gp120 CN54 as a function of immobilization density.

	Density of gp120 CN54 (ng/mm^2^)		
	0.2	1	4.8
	***k***_**a**_ **, M** ^**−1**^ **s** ^**−1**^ **(×10** ^**4**^ **)**		
HJ16	12.6 ± 0.05	15.3 ± 0.04	16.6 ± 0.03
NIH45-46 G54W	13.3 ± 0.05	14.9 ± 0.04	13.8 ± 0.03
2-1262	28 ± 0.16	33.1 ± 0.17	31.7 ± 0.17
b12	173 ± 1.6	230 ± 0.9	120 ± 0.4
CH103	2.4 ± 0.04	2.8 ± 0.01	2.9 ± 0.01
	***k***_**d**_ **, s** ^**−1**^ **(×10** ^**−4**^ **)**		
HJ16	0.85 ± 0.019	0.13 ± 0.014	0.006 ± 0.003
NIH45-46 G54W	0.35 ± 0.07	0.024 ± 0.008	0.02 ± 0.008
2-1262	1.12 ± 0.19	1.14 ± 0.15	0.31 ± 0.04
b12	38 ± 0.34	31.4 ± 0.15	12 ± 0.11
CH103	20.5 ± 0.29	8.13 ± 0.11	1.65 ± 0.04
	**K**_**D**_ **, nM**		
HJ16	0.67	0.08	0.004
NIH45-46 G54W	0.26	0.01	0.01
2-1262	0.39	0.34	0.1
b12	2.19	1.36	1.00
CH103	85.8	28.5	5.65

All association rate constants were determined by global analyses of real-time binding, using Langmuir kinetic model. The dissociation rate constants of Abs b12 and CH103 were determined also by global analyses, whereas these constants for Abs HJ16, NIH45-46 G54W and 2-1262 were determined by separate Langmuir dissociation model at Ab concentrations of 100 nM in case of 2-1262, and 50 nM in cases of HJ16 and NIH45-46 G54W. The equilibrium dissociation rate constants were determined from the ration *k*_d_/*k*_a_. Standard errors of association and dissociation rate constants were derived by Biaevaluation software (global Langmuir analyses). In cases of separate determination of dissociation (Abs HJ16, NIH45-46 G54W and 2-1262) errors were determined by three independent fits of experimental data.

**Figure 4:**
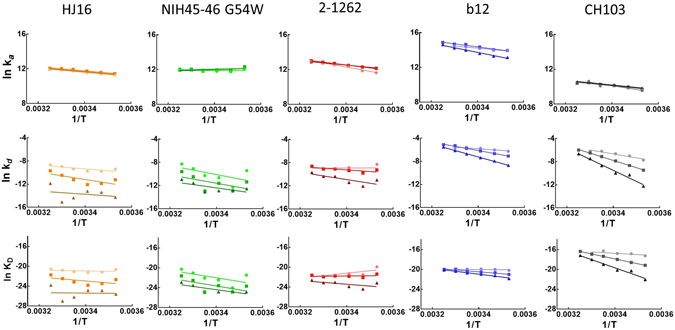
Temperature dependences of binding kinetics and of K_D_ of Abs specific for CD4-binding site of gp120 – HJ16 (orange), NIH45-46 G54W (green), 2-1262 (red), b12 (blue) and CH103 (black). The different intensities of colors correspond to differences in the immobilization density of gp120 CN54, namely pale: 0.2 ng/mm^2^, median: 1 ng/mm^2^ and intense: 4.8 ng/mm^2^. The Arrhenius plots were generated by linear regression analyses. The values of R^2^ of the presented fits are as follows: (1) HJ16, *k*_a_, low density (l.d.): 0.9856, median density (m.d.): 0.9731 and high density (h.d.): 0.9661; *k*_d_, l.d.: 0.5940, m.d.: 0.5581 and h.d.: 0.06745; K_D_, l.d.: 0.07256, m.d.: 0.3175 and h.d.: 0.001676. (2) NIH45-46 G54W, *k*_a_, l.d.: 0.01817, m.d.: 0.1816 and h.d.: 0.3288; *k*_d_, l.d.: 0.3359, m.d.: 0.3242 and h.d.: 0.4766; K_D_, l.d.: 0.3754, m.d.: 0.4143 and h.d.: 0.6340. (3) 2-1262, *k*_a_, l.d.: 0.9635, m.d.: 0.9978 and h.d.: 0.9983; *k*_d_, l.d.: 0.001413, m.d.: 0.5753 and h.d.: 0.6117; K_D_, l.d.: 0.5864, m.d.: 0.03338 and h.d.: 0.3580. (4) b12, *k*_a_, l.d.: 0.9690, m.d.: 0.9632 and h.d.: 0.9806; *k*_d_, l.d.: 0.8443, m.d.: 0.9924 and h.d.: 0.9987; K_D_, l.d.: 0.06348, m.d.: 0.9438 and h.d.: 0.9786. (5) CH103, *k*_a_, l.d.: 0.8742, m.d.: 0.9373 and h.d.: 0.9772; *k*_d_, l.d.: 0.9254, m.d.: 0.9982 and h.d.: 0.9769; K_D_, l.d.: 0.4867, m.d.: 0.9994 and h.d.: 0.9706.

We then studied the temperature dependence of the kinetic rate constants of the Abs to CD4bs at varying gp120 densities. Variations in temperature from 10 to 35 °C (presented as reciprocal values in Kelvin), had a little impact on the association rate constants of all Abs, irrespective of the surface density of the target antigen (Fig. 4). Most of the Abs slightly increased their association rates with an elevation of the interaction temperature. In contrast to the association kinetics, temperature changes profoundly impacted dissociation kinetics. Reduction of surface density of gp120 resulted in a marked decrease of *k*_d_ constant for all studied Abs. However, temperature affected most profoundly the dissociation rate of low affinity Ab CH103, as evident by a substantial increase in the slope of the Arrhenius plots. It is noteworthy that a similar relationship between antigen density and temperature sensitivity of binding kinetics was detected in the case of 447-52D and b12 Abs (Fig. 2).

Importantly, with an increase of the density of immobilized gp120, there was a concomitant increase of the temperature dependency of binding affinity of low affinity Ab CH103 (Fig. 4). Variations of equilibrium dissociation constants of studied Abs closely reflected the variation of kinetic rates of dissociation.

**Figure 5:**
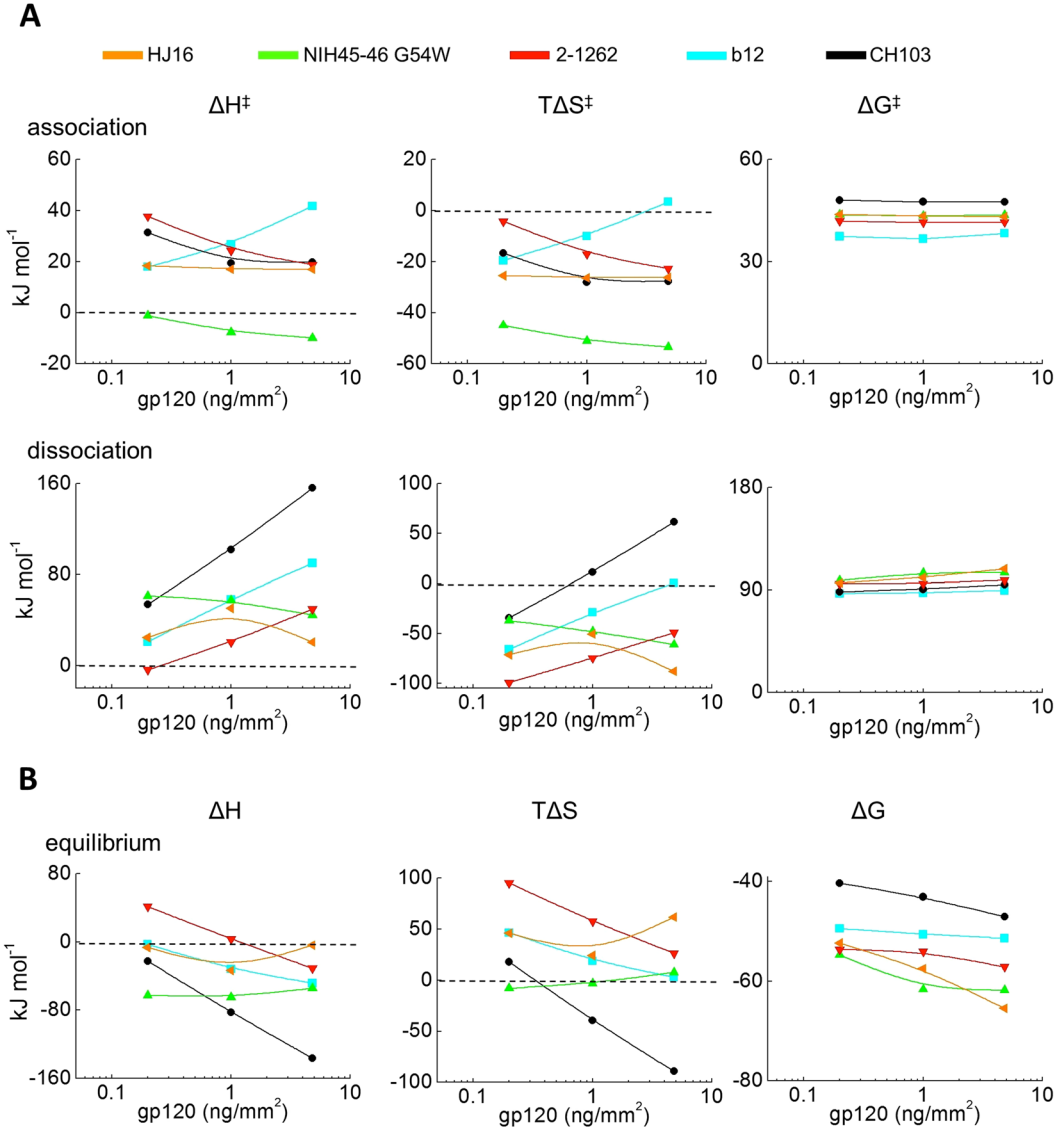
(**A**) Effect of density of surface-bound antigen on the activation thermodynamics of gp120 CD4-binding site specific Abs. Changes of thermodynamic parameters of HJ16 (orange line and symbols), NIH45-46 G54W (green line and symbols), 2-1262 (red line and symbols), b12 (blue line and symbols) and CH103 (black line and symbols) as a function of surface density of gp120 CN54 are presented. The values of the thermodynamic parameters were calculated by Eyring’s analyses using slopes of Arrhenius plots depicted on Fig. 4. All thermodynamic parameters were calculated at a reference temperature of 25 °C (293.7 K). (**B**) Effect of density of surface-bound antigen on the equilibrium thermodynamics of CD4 binding site-specific Abs. The values of the thermodynamic parameters were evaluated as described in the experimental procedures. All equilibrium thermodynamic parameters were calculated at a reference temperature of 25 °C (293.7 K).

Arrhenius plots depicted on Fig. 4 were used to calculate the values of activation and equilibrium thermodynamics of HIV-1 Abs. The association of Abs to gp120 was not associated with qualitative changes in the activation enthalpy and entropy (except in the case of TΔS^‡^ for b12). In addition, extend of the quantitative changes in the energies as a function of antigen density were limited, reaching maximally 21 kJ mol^−1^ and 22 kJ mol^−1^ in the case of association enthalpy and entropy, respectively for b12 (Fig. 5A). In contrast, substantial changes in the thermodynamic parameters as a function of the antigen density were observed during the dissociation phase, especially in the case of the two Abs (CH103 and b12) that recognize gp120 CN54 with the lowest binding affinities. Thus, an increase in antigen density of 25 folds resulted in an augmentation of positive value of the dissociation enthalpy of CH103 by approximately 100 kJ mol^−1^. The density of immobilized antigen had also a considerable effect on the dissociation entropy of this Ab, characterized by an increase in TΔS^‡^ values by approximately 95 kJ mol^−1^ (Fig. 5A). Importantly, in addition to the quantitative changes in binding energy, antigen density qualitatively influenced the entropy change of the low affinity Ab. Thus, at low antigen density, the TΔS^‡^ value of CH103 had a negative value, which was gradually transformed to positive as the antigen density increased (Fig. 5A).

Analyses of equilibrium thermodynamic parameters as a function of the antigen density also demonstrated that the most pronounced changes in binding energies occur for the interaction of the low affinity Ab CH103 (Fig. 5B). Thus, an increase of antigen density was associated with a more favorable value of ΔH°, with an increase >110 kJ mol^−1^. Substantial changes of equilibrium entropy values were detected as well. This parameter was slightly favorable at lowest antigen density but become highly unfavorable (decreasing by 106 kJ mol^−1^) when the density of gp120 was elevated. Interestingly, despite profound changes in enthalpy and entropy of CH103, the changes of equilibrium free energy of Gibbs were less affected by alterations in antigen density (Fig. 5B). This result may be explained by enthalpy-entropy compensation effect^17^ that was the most evident for the interaction of CH103 with gp120.

In summary, data from non-equilibrium and equilibrium thermodynamic analyses of anti-CD4bs antibodies are in full agreement with the data obtained with gp120 IIIB and b12 and 447-52D Abs. Indeed, data from both series of experiments indicate that low affinity Abs experience the most considerable impact on antigen recognition mechanism as result of changes in density of surface-bound antigens.

## Discussion

In the present study, we analyzed the impact of antigen density on the binding energetics of human Abs. We evaluated the molecular recognition of the HIV-1 envelope protein gp120 (strains IIIB and CN54) by neutralizing IgGs. The Abs studied here differed by at least three orders of magnitude in the values of their binding affinities to gp120. Our data reveal that the extent and the nature of the changes on the binding energetics as a function of antigen density strongly depend on the binding affinity of Abs. Thus, Abs that recognize gp120 with lower affinities experience considerable qualitative and quantitative transformation in the binding energetics as surface antigen density increases. In contrast, Abs with high affinities were considerably less sensitive under the same conditions. The observed changes of binding behavior with high affinity Abs predominantly affected binding kinetics but not thermodynamics; these changes are consistent with a bias on the binding process of mass transport that is associated with an increase in ligand density^14, 16^.

Important qualitative and quantitative changes in the activation and equilibrium thermodynamics observed for 447-52D and CH103 binding to surfaces with different density of antigen can be explained by the involvement of different molecular mechanisms when the Ab recognizes low and high densities of antigen. Changes in enthalpy and entropy describe different aspects of the intermolecular recognition process. Thus, enthalpy changes, depend on the net-quantity and on the type of non-covalent interactions formed between interacting proteins or between proteins and the solvent. The changes in the entropy reflect alterations in the disorder of the system that may be driven by restriction of conformational freedom of binding surfaces or reconfiguration of the solvent structure^18–21^. The transformation of an entropy-driven to an enthalpy-driven process in the case of interactions of low affinity Abs with an increase of gp120 density implies that, high antigen density is associated with a considerable restriction of the structural freedom of IgG (negative contribution to TΔS). The effect described here may be a quantitative reflection of the occurrence of binding phenomena that are characteristic for the IgG class, and might take place at surfaces such as – avidity enhancement *via* bivalent antigen binding^8, 22, 23^, formation of non-covalently bound IgG hexamers^11^, or IgG “bipedal” stochastic movements on antigenic surfaces^10^. These processes would be influenced by the pattern of distribution of epitopes. For example, high antigen density may facilitate the formation and stabilization of non-covalently bound IgG hexamers – such a process will have strong negative impact on the entropy, as disorder in the system will be considerably reduced upon formation of the supramolecular complexes. Importantly, IgG hexamer can bind other hexamers through lateral Fab-Fab interactions, thus forming extensive two-dimensional lattices^24^. Another process - IgG dynamics on antigen surfaces (“IgG walking”) - strongly depends on the bivalency of the molecule and is therefore influenced by surface antigen density^10^. One can hypothesize that engagement of Ab in a stochastic two-dimensional walk on the antigen surfaces occurs only above a particular threshold of antigen density; too distant antigens would not necessary allow the involvement of both Fab fragments for antibody binding. This process can also be expected to lead to a reduction of the disorder in the system – Abs will have restrictions in their dynamics from the three-dimensional to the two-dimensional space resulting in negative values of the equilibrium entropy.

Interestingly, the Abs with the highest affinity used in this study (b12 in case of gp120 IIIB; NIH45-46 G54W, 2-1262, and HJ16 in case of CN54) conserved their thermodynamic behavior at varying antigen densities. This may be explained by the negligible dissociation rate, which is a characteristic feature for these Abs. Thus, the limited dissociation from bound epitope would impair any antigen surface-dependent phenomenon that requires binding dynamics of IgG molecules. Thus, our data strongly imply that Abs with lower affinities have a higher dynamic potential and can explore alternative mechanisms for sampling antigens. The biological significance of the transformation in binding mechanism of Abs may be considerable as the formation of supramolecular complexes of IgG were demonstrated to be the form that activates the complement system on antigenic surfaces^11^. Importantly, IgGs engineered to have enhanced hexamer formation were shown to activate complement irrespective of antigen density^13^. In contrast, normal IgGs activate the complement only at high antigen densities^13^.

Ab avidity enhancement effect strongly depends on the density of antigens, since the reach of the two binding sites of IgG is about 150 Å^8, 9^. Engagement of both binding sites result in a marked increase in binding affinity, primarily through a reduction in the values of *k*_d_. By using mathematical modeling, it was recently predicted that avidity enhancement due to bivalent IgG binding have little contribution in the case of high affinity Abs^23^. In contrast, low affinity binding Abs can considerably augment their functional affinity as result of a bivalent antigen engagement^23^. It has been proposed that the avidity effect is an important factor conferring to IgG the ability to resist the deleterious effect of mutations within the targeted epitopes^9^. Low density of envelope proteins of HIV-1^25^ combined with high mutation rate was hypothesized to play an important role in the evasion from Ab-mediated neutralization^9^. In this regard, polyreactivity of virus-specific antibodies (regardless of their neutralization potential) was proposed to favor bivalent binding to virions expressing low density surface antigens by heteroligation, thus overcoming the absence of homotypic bivalent binding to Env proteins^26, 27^.

The considerable effect of antigen density on the recognition mechanisms of Abs with distinct affinities, suggests that this phenomenon plays a role in the regulation of the effector functions of IgG. Further studies are required to fully appreciate the biological consequences of the effect of antigen density on antibody binding mechanisms.

## Methods

### Materials

Broadly neutralizing HIV-1 Abs - b12, NIH45-46 G54W, HJ16, and 447-52D and recombinant gp120 proteins (HIV-1 variants IIIB, clade B and CN54, clade C) were kindly provided by National Health Institute AIDS Research and Reagents Program. 2-1262^28^ and CH103^29^ were produced as recombinant monoclonal IgG1 antibodies by co-transfection of 293T or 293F cells as previously described^30^. Antibodies were purified by batch/gravity-flow affinity chromatography using protein G sepharose 4 fast flow beads (GE Healthcare).

### Real time interaction analyses

Kinetics of monoclonal Abs binding to gp120 was evaluated by surface plasmon resonance-based optical biosensor system – Biacore 2000 (Biacore, GE Healthcare, Uppsala, Sweden). CM5 sensor chips (Biacore, GE Healthcare) were used throughout the study. Recombinant HIV-1 gp120 IIIB or gp120 CN54 were covalently immobilized using amine-coupling kit (Biacore, GE Healthcare). Briefly, gp120 proteins were diluted in 5 mM maleic acid (pH 4) to final concentrations of 20 μg/ml or 50 μg/ml and injected over sensor surfaces activated by a mixture of 1-Ethyl-3-(3-dimethylaminopropyl)-carbodiimide (EDC)/N-hydroxysuccinimide (NHS). The optimal pH of immobilization solution was chosen after an assessment of pre-concentration of gp120 to the carboxymethylated dextran of sensor surface, using maleic acid solutions with pH 4, 5 or 6. Uncoupled activated carboxyl groups were blocked by injection of 1 M solution of enthanolamine.HCl. Differences in the resonance response in the range of 100 to 6000 RU (corresponding to range of 0.1–6 ng/mm^2^ immobilized protein) were achieved by varying the contact time of gp120 containing solutions with the pre-activated sensor surfaces. A control surface was prepared on each chip by EDC/NHS - activation and subsequent deactivation of the carboxyl groups with excess (1 M) of ethanolamine.HCl. The running buffer HBS-EP (10 mM HEPES pH 7.2; 150 mM NaCl; 3 mM EDTA, and 0.005% Tween 20) was filtered through 0.22 µm filter and degassed under vacuum, and was used during the immobilization and binding analyses.

To evaluate the binding kinetics of the interactions of 447-52D and b12 with gp120, Abs were serially diluted (two-fold each step) in HBS-EP to concentrations ranging from 50 to 0.097 nM (447-52D) and 25 to 0.048 nM (b12) and injected over immobilized gp120 IIIB at various densities. The interaction of CD4-binding site specific Abs with gp120 CN54 were measured after serial dilutions of 50–0.039 nM (HJ16 and NIH45-46 G54W), 10–0.078 nM (b12), 200–1.562 nM (CH103) and 100–0.78 nM (2–1262). The flow rate during all interaction analyses was set at 30 µl/min. The association and dissociation phases of the binding of Abs were monitored for 4 and 5 min, respectively. The sensor chip surfaces were regenerated by exposure to a solution of 4 M MgCl_2_ for 30–60 sec. Kinetic measurements were performed as a function of temperature in the 10–35 °C range. The evaluation of the kinetic data was performed by BIAevaluation version 4.1.1 Software (Biacore) by applying Langmuir binding model.

### Evaluation of binding thermodynamics

The evaluation of the thermodynamic parameters was performed as described before^31, 32^. In brief, we used Eyring’s analyses for evaluation of the activation thermodynamics of the interactions of Abs with different densities of gp120. To this end the kinetic rate constants obtained at distinct temperatures (15, 20, 25, 30, 35 °C in cases of b12 and 447-52D binding to gp120 IIIB and at 10, 15, 20, 25, 30, 35 °C in cases of interactions of HJ16, NIH45-46 G54W, b12, 2-1262 and CH103 with gp120 CN54) were used to build Arrhenius plots. The values of the slopes of the Arrhenius plots were calculated by applaying linear regression analysis by using GraphPad Prism v.6 (GraphPad Prism Inc. USA) and were substituted in the equation -

1 $$Ea=-slope\times R,$$

Where the

2 $$ \mbox{``} \mathrm{slope}\mbox{''}=\partial \,\mathrm{ln}({{\rm{k}}}_{a/d}/\partial (1/T))$$

where *Ea* is the activation energy. The changes in enthalpy, entropy and Gibbs free, characterizing the association and dissociation phases were estimated by using the equations:

3 $$\Delta {H}^{\ddagger}={\rm{Ea}}-{\rm{RT}}$$

4 $$\mathrm{ln}({{\rm{k}}}_{{\rm{a}}/{\rm{d}}}/{\rm{T}})=-{\rm{\Delta }}{H}^{\ddagger}/\mathrm{RT}+\Delta {S}^{\ddagger}/R+\,\mathrm{ln}(k^{\prime} /h)$$

5 $$\Delta {G}^{\ddagger}=\Delta {H}^{\ddagger}-{\rm{T}}\Delta {S}^{\ddagger}$$

where T is the temperature in Kelvin degrees, *k′* is the Boltzman constant and *h* is the Planck’s constant.

The equilibrium values of the thermodynamic parameters were calculated using the equations:

6 $${\rm{\Delta }}{G}_{{\rm{e}}{\rm{q}}}={\rm{\Delta }}{G}_{a}^{\ddagger}-{\rm{\Delta }}{G}_{d}^{\ddagger},$$

7 $$\Delta {H}_{eq}=\Delta {H}_{a}^{\ddagger}-\Delta {H}_{d}^{\ddagger},$$

8 $${\rm{T}}\Delta {{\rm{S}}}_{{\rm{eq}}}=T\Delta {S}_{{\rm{a}}}^{\ddagger}-{\rm{T}}\Delta {{{\rm{S}}}^{\ddagger}}_{{\rm{d}}}$$

The equilibrium thermodynamic parameters were also calculated by using van’t Hoff equation. Activation and equilibrium thermodynamic parameters were determined at reference temperature of 25 °C (298.15 K).

### Statistical analyses

Linear regression analyses were performed with Graph Pad v. 6 softwere (GraphPad Software, La Jolla, CA). The fits with *p* < 0.05 were considered significant. Kinetic data were expressed as mean ± s.e.m. Standard errors were calculated with Biaevaluation v 4.1.1 (Biacore).

## Electronic supplementary material

Supplementary Figure 1
