# Supplementary material for: Impact of Antigen Density on the Binding Mechanism of IgG Antibodies
Source: Sci Rep. 2017 Jun 19;7:3767. doi: 10.1038/s41598-017-03942-z (PMC5476644; doi:10.1038/s41598-017-03942-z)
Supplement: Supplementary file 1 — Supplementary Figure 1 [file 41598_2017_3942_MOESM1_ESM.pdf]

Impact of Antigen Density on the Binding Mechanism of IgG Antibodies

Maya Hadzhieva[1,2,3,4], Anastas D. Pashov[1,2,3,4], Srinivas Kaveri[2,3,4], Sébastien Lacroix-Desmazes[2,3,4], Hugo Mouquet[5] and Jordan D. Dimitrov[2,3,4]

[1]Institute of Microbiology, Bulgarian Academy of Sciences, 1113 Sofia, Bulgaria;

[2]Sorbonne Universités, UPMC Univ Paris 06, UMR_S 1138, Centre de Recherche des Cordeliers, 75006 Paris, France;

[3]INSERM, UMR_S 1138, Centre de Recherche des Cordeliers, 75006 Paris, France;

[4]Université Paris Descartes, Sorbonne Paris Cité, UMR_S 1138, Centre de Recherche des Cordeliers, 75006 Paris, France;

[5]Laboratory of Humoral Response to Pathogens, Department of Immunology, Institut Pasteur, and INSERM U1222, 75015 Paris, France.

Supplemental Figure 1

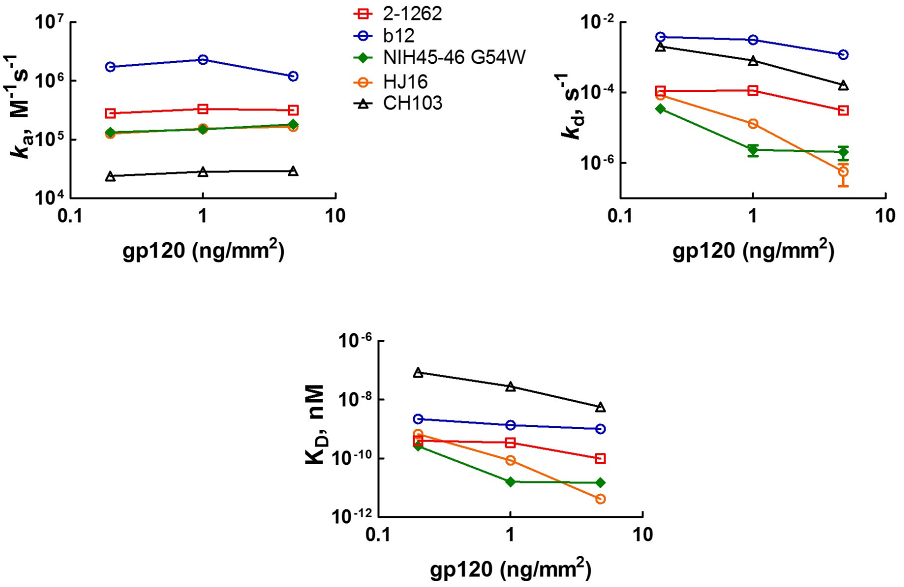

Supplemental Figure 1. Effect of density of surface immobilized gp120 CN54 on the association rate constant; dissociation rate constant, and equilibrium dissociation constant of CD4bs human monoclonal IgG antibodies. The binding kinetics was measured at 25 °C.
